# Ocean Acidification Disrupts Prey Responses to Predator Cues but Not Net Prey Shell Growth in *Concholepas concholepas* (loco)

**DOI:** 10.1371/journal.pone.0068643

**Published:** 2013-07-03

**Authors:** Patricio H. Manríquez, María Elisa Jara, María Loreto Mardones, Jorge M. Navarro, Rodrigo Torres, Marcos A. Lardies, Cristian A. Vargas, Cristian Duarte, Stephen Widdicombe, Joseph Salisbury, Nelson A. Lagos

**Affiliations:** 1 Instituto de Ciencias Marinas y Limnológicas, Laboratorio de Ecología y Conducta de la Ontogenia Temprana (LECOT) and Laboratorio Costero de Recursos Acuáticos de Calfuco. Facultad de Ciencias, Universidad Austral de Chile, Valdivia, Chile; 2 Centro de Investigación en Ecosistemas de la Patagonia (CIEP), Coyhaique, Chile; 3 Facultad de Artes Liberales, Universidad Adolfo Ibáñez, Santiago, Chile; 4 Laboratorio de Funcionamiento de Ecosistemas Acuáticos (LAFE), Unidad de Sistemas Acuáticos, Centro de Ciencias Ambientales EULA, Universidad de Concepción, Concepción, Chile; 5 Facultad de Ecología y Recursos Naturales, Departamento de Ecología y Biodiversidad, Universidad Andrés Bello, Santiago Chile; 6 Plymouth Marine Laboratory, Prospect Place, West Hoe, Plymouth, Devon, United Kingdom; 7 Ocean Processes Analysis Lab, University of New Hampshire, Durham, New Hampshire, United States of America; 8 Facultad de Ciencias, Universidad Santo Tomás, Ejercito, Santiago, Chile; Institute of Marine Research, Norway

## Abstract

**Background:**

Most research on Ocean Acidification (OA) has largely focused on the process of calcification and the physiological trade-offs employed by calcifying organisms to support the building of calcium carbonate structures. However, there is growing evidence that OA can also impact upon other key biological processes such as survival, growth and behaviour. On wave-swept rocky shores the ability of gastropods to self-right after dislodgement, and rapidly return to normal orientation, reduces the risk of predation.

**Methodology/Principal Findings:**

The impacts of OA on this self-righting behaviour and other important parameters such as growth, survival, shell dissolution and shell deposition in *Concholepas concholepas* (loco) were investigated under contrasting *p*CO_2_ levels. Although no impacts of OA on either growth or net shell calcification were found, the results did show that OA can significantly affect self-righting behaviour during the early ontogeny of this species with significantly faster righting times recorded for individuals of *C. concholepas* reared under increased average *p*CO_2_ concentrations (± SE) (716±12 and 1036±14 µatm CO_2_) compared to those reared at concentrations equivalent to those presently found in the surface ocean (388±8 µatm CO_2_). When loco were also exposed to the predatory crab *Acanthocyclus hassleri*, righting times were again increased by exposure to elevated CO_2_, although self-righting times were generally twice as fast as those observed in the absence of the crab.

**Conclusions and Significance:**

These results suggest that self-righting in the early ontogeny of *C. concholepas* will be positively affected by *p*CO_2_ levels expected by the end of the 21st century and beginning of the next one. However, as the rate of self-righting is an adaptive trait evolved to reduce lethal predatory attacks, our result also suggest that OA may disrupt prey responses to predators in nature.

## Introduction

A wide range of marine organisms, including phytoplankton, invertebrates and fish, synthesize some form of calcium carbonate structure. The most conspicuous of these structures are the skeletons of corals, molluscs, coccolithophores, and crustaceans. Ocean acidification (OA), caused by the rapid uptake of anthropogenic CO_2_ into the surface ocean, is a term, which describes the currently observed reduction in seawater pH and carbonate ion concentration (CO_3_^2−^) [1]. In turn, these changes in seawater chemistry are widely predicted to not only decrease calcium carbonate (CaCO_3_^2−^) formation in many marine organisms, but also possibly accelerate its dissolution and/or erosion [2]. This idea is supported by studies in which the carbonate structures in a number of marine invertebrates have been shown to decrease in size in response to OA [3], [4]. However, recent evidence has also shown that carbonate structures of fish [5], [6] and some invertebrates [7], [8] can actually increase in size as a homeostatic response to changing internal levels of CO_2_. Therefore, regardless of whether OA induces an increase or decrease in carbonate production, it is evident that the calcified structures of many marine invertebrates could be affected significantly by the chemical changes associated with on-going OA. Although most research on OA has largely focused on the process of calcification and the physiological trade-offs employed by calcifying organisms to support the building of calcium carbonate structures [9]–[11], there is growing evidence that OA can also impact upon other key biological processes such as survival, growth, behaviour and metabolism [12], [13]. In addition, recent studies have indicated that OA can also have fundamental effects on predator-prey interactions or behavioural traits [14]–[23].

Several studies have suggested that measuring the righting time, how long it takes an individual to return to its normal orientation after having been disturbed, could provide a good indication of whether an individual is exhibiting a stress related response [24]–[28]. Such a stress response could be elicited by a variety of different mechanisms including the direct impacts of a physical or chemical change (e.g. temperature, pH and pollution), a visual stimuli (e.g. shadow or sight of predator) or detection of a chemosensory cue. In marine gastropods, chemoreception of odour cues emanating from food items, conspecifics, or predators involves the osphradium, an external sensory organ containing neuro-epithelial cells which monitor the physiochemical properties of the surrounding seawater [29], [30]. Therefore, the osphradium can be considered as the primary organ acquiring environmental information and likely plays an important role in modifying the response of an individual to environmental stressors. Evidence from fresh water snails has indicated that the sensorial capacity of the osphradium is affected by low environmental calcium [31]. Similarly, juvenile fishes become more active and attracted to stimuli they normally avoid when reared under elevated CO_2_ conditions [16]. Such changes have been attributed to the disruption of neurological function caused by exposure to high CO_2_ levels [13], [18], [32]. This suggests that future reductions in the pH of seawater, as a consequence of OA, might also have implications for other important behavioural traits mediated by chemical perception such as settlement on appropriate habitat, prey detection, predator avoidance and mate selection.

In Chile, the gastropod *Concholepas concholepas* is both an economically and ecologically important species inhabiting subtidal and intertidal marine habitats [33], [34]. By inhabiting rocky intertidal environments *C. concholepas* is often exposed to highly turbulent conditions and when exposed to strong wave action this species adheres tightly to the rock surface with their foot [35]. However, when feeding and handling prey items, the foot of *C. concholepas* is often removed from the rock leaving it vulnerable to being dislodged. In addition, the dislodgement or falling reflex in *C. concholepas* has been described as an important escape tactic when the individuals adhered to inclined rocks in nature, or to the walls of aquarium in captivity, are subject to mechanical disturbance [35]–[36]. This behaviour indicates that self-righting speed and alarm response to predator cues could be important behavioural traits under selection for species living in turbulent, predatory rich environments and particularly for *C. concholepas*. Preliminary work by Manríquez (unpublished) has indicated that delayed metamorphosis in *C. concholepas* reduces the speed with which early post-metamorphic individuals can return to their normal position after being placed upside-down. One consequence of this is that these smaller individuals of *C. concholepas* (ca 0.2 cm) are more likely to be exposed to visual predators such as crabs than larger, faster developing individuals. Similarly, chemically mediated behavioural responses in terrestrial and marine gastropods indicate that they can discriminate between environmental odours originating from several different sources such as food, damaged conspecifics, and predators [25], [37]. Under laboratory conditions *C. concholepas* responded negatively to the odours of predatory crabs and starfish, avoiding crawling displacement in the direction of the odour source [38]. Moreover, small juveniles of *C. concholepas* are also able to achieve cryptic shell colouration and avoid lethal attacks by crabs [39].

Early life stages are considered to be more susceptible to external environmental changes than later stages due to their relatively higher surface to area/volume ratio and lower ability to compensate for changes in internal acid-base balance [40]. Recent information has shown that under low pH conditions decision making is disrupted in both hermit crabs [18] and fish [13]–[15], [41]. This suggests that future reductions in the pH of seawater, as a consequence of OA, may have implications for other important behavioural traits such as settlement on appropriate habitat, prey detection, predator avoidance, and mate selection; all important functions associated with the possible role of the osphradium in *C. concholepas* [42]. Since prey perception and shell formation can be affected by pH conditions and CO_3_^2−^ availability, the present study used small individuals of *C. concholepas* as a model for testing simultaneously the potential impacts of OA on those behavioural traits associated with righting and the perception of predation risk, together with other relevant and connected processes such as growth and net calcification.

## Materials and Methods

### Collection and Rearing of Experimental Animals

#### Ethics statement

The model species used in the present study is not an endangered species (IUCN Red Data Books) and is not subject to restrictions under Chilean legislation; therefore specific permission for their collection from natural habitats was not required. Moreover, the individuals were collected from an open access shore and therefore no special authorization from a land or shore owner were required.

Small individuals of *C. concholepas* with a maximal length at the margin of the shell aperture of 1.0 cm (i.e. peristomal length) were collected during the spring low tides of June 2011 from a rocky intertidal platform in Antofagasta, northern Chile. According to the literature, sexual maturity in *C. concholepas* is achieved when an individual’s peristomal length exceeds 4 cm [43]. Therefore, all the experimental individuals of *C. concholepas* used in the present study can be considered as juvenile individuals in their early ontogeny. After collection, the individuals were transported to the Laboratorio Costero de Recursos Acuáticos at Calfuco on the coast near Valdivia, southern Chile, where all the experiments were conducted. Individuals were reared in natural seawater in a Plexiglas aquarium for 1 month to acclimatize to laboratory conditions, fed *ad libitum* with small individuals of the mitilid *Perumitylus purpuratus*, and water changes were conducted every second day. After this first phase (from this point on referred to as the acclimation phase), when individuals were between 1.5 and 2.1 cm in size, groups of 10 individuals were randomly assigned to one of the three treatment conditions in preparation for the second phase of the study (from this point on referred to as the acidification phase). The *p*CO_2_ considered for the medium (716 µatm) and high (1036 µatm) levels were chosen to represent conditions predicted under worst-case scenarios for the end of the 21st century and the beginning of the next one [44]. To avoid cannibalism during the acidification phase each individual was maintained separately in 0.5 L plastic exposure containers, which were filled with the appropriate (either 388, 716 or 1036 µatm) pre-conditioned seawater. During this 83 day acidification phase, the seawater in each of the rearing containers was replaced daily with fresh, 0.5 µm filtered seawater which had been conditioned to the appropriate *p*CO_2_ level. To ensure that *p*CO_2_ levels remained stable within the exposure containers between each daily water change, the exposure containers were sealed with a plastic lid with a small hole to allow tube access. Inside each exposure container, the end of each tube was fitted with a fine plastic tip through which a continuous stream of either air (388 µatm CO_2_) or enriched CO_2_ air (716 or 1036 µatm) was bubbled. The enriched CO_2_ air was created using the methods described by Navarro et al. [45]. This continuous dosing allowed the required levels of *p*CO_2_ to be precisely maintained during the acidification phase of this study (see Table 1). A second hole in the lid of the exposure containers was used as air outlet to prevent any build-up of pressure within the container. Throughout the acidification phase, small individuals of the mussel *P. purpuratus* were also provided *ad libitum* as food, up until the experimental measurements began. Whilst the experimental measurements were being taken the exposure containers were semi-immersed in a water bath with running seawater in order to maintain the temperature ca. 12±1°C. In rocky intertidal habitats along the Chilean coast the predatory crab *Acanthocyclus hassleri* prey on barnacles and mussels [46] and small individuals of *C. concholepas* [38]. Since individuals of *C. concholepas* were originally collected in the field, where the predatory crab *A. hassleri* is currently found [38], [46], for the purpose of the present study loco were considered to be predator experienced.

**Table 1:** Average (± SE) conditions of the seawater used to maintain *C. concholepas* during the acidification phase (July to October 2011).

CO_2_ system parameters	Experimental *p*CO_2_ levels (µatm)		
	388	716	136
pH@25°C (pH units)	7.837 (0.008)	7.615 (0.008)	7.469 (0.006)
pH *in situ* (pH units)	8.029 (0.009)	7.796 (0.008)	7.638 (0.006)
Salinity (psu)	30.89 (0.36)	31.35 (0.35)	31.48 (0.34)
Temperature (°C)	11.39 (0.24)	11.39 (0.23)	11.46 (0.23)
TA (µmol Kg^−1^)	2118.19 (20.13)	2121.58 (20.35)	2127.21 (21.03)
*p*CO_2_ *in situ* (µatm)	387.92 (7.94)	715.86 (12.41)	1036.04 (14.49)
[CO_3_^2−^] *in situ* (µmol Kg^−1^)	118.36 (3.45)	72.64 (1.95)	53.06 (1.36)
Ωaragonite	1.82 (0.05)	1.12 (0.03)	0.82 (0.02)
Ωcalcite	2.88 (0.08)	1.77 (0.05)	1.27 (0.03)

pH (total scale), Total alkalinity (TA in µmol kg^−1^), partial pressure of CO_2_ (levels of pCO_2_ in µatm), Carbonate ion concentration (CO_3_^2−^ in µmol kg^−1^), saturation states of the water in terms of aragonitic and calcite minerals (Ω_aragonite_ and Ω_calcite_ respectively). The different experimental levels of *p*CO_2_ in the mesocosms and in the rearing containers were achieved and maintained during the entire experimental period by active injection of CO_2_ and air (see Materials and Methods for further details).

¥ Based on rate of change in pH predicted by the most extreme scenario (RCP8.5 scenario) of atmospheric CO_2_. See Meinshausen et al. 2011.

### Generation of CO_2_ Enriched Air and Verification of *p*CO_2_ in air-CO_2_ Mixtures

The seawater acidification unit provided conditioned seawater at acidification (*p*CO_2_) levels of 388±8 µatm (atmospheric level), 716±12 and 1036±14 µatm. Three large (250 litres), independent plastic reservoir tanks were filled with filtered (0.5 µm) seawater (FSW). The seawater was continuously bubbled with either ambient air (approximately 380 µatm CO_2_) or enriched CO_2_ air (716 or 1036 µatm), which had been premixed following precisely the method described by Navarro et al. (2013) [45] to create the required mixed gas. Three times a week seawater samples were taken from each unit to assure consistency in carbonate parameter measurements (Table 1). The pure air bubbling in the 250 L container yielded a *p*CO_2_ of 388±8 µatm and was used as control; for CO_2_ enriched treatments, air was blended with pure CO_2_ using Mass Flow Controllers (MFCs, www.aalborg.com) to produce dry air-CO_2_ mixtures of approximately 750 and 1200 µatm; this blend was then bubbled into the corresponding reservoir tank yielding 716±12 and 1036±14 µatm, respectively. The discrepancies between the measured *p*CO_2_ in CO_2_-air mixture and the actual *p*CO_2_ in the seawater at the reservoirs tanks may be attributed to: (1) the reduction in *p*CO_2_ of the air-CO_2_ mixture after become saturated in water during the bubbling; and (2) an incomplete equilibrium of the seawater and air-CO_2_ mixtures, particularly at highest CO_2_-air mixture treatment (i.e. 1200 µatm, Table 1). Every day the reservoir tanks were topped up with treated FSW and once a week the tanks were cleaned and the total water content of each reservoir tank was replaced with fresh seawater, and normally after 5–12 hr of vigorous CO_2_-air mixture bubbling (an air flow ranging between 2000–3000 ml min^−1^) the seawater *p*CO_2_ reaches the reported mean values. During the experiment, seawater pH, temperature, salinity and total alkalinity were measured in each reservoir every three days in order to determine the actual carbonate system speciation in the equilibrated water. Clean dry air was generated by compressing atmospheric air (117 psi) using an oil-free air compressor and passed through particle filters (1 µm) to remove particulates. The clean air flow was set to 5 liter min^−1^ for both treatments using air MFCs. Downstream of the air MFCs mix with pure CO_2_ gas which flow was manually adjusted to produce air-CO_2_ mixtures *p*CO_2_ in dry air of 750 µatm and 1200 µatm, respectively. The air-CO_2_ mixtures *p*CO_2_ levels were measured using a CO_2_ analyzer (QUBIT SYSTEMS S151 CO_2_ Analyzer calibrated with CO_2_-free air and a standard CO_2_-air mixture of 1110 µatm provided by INDURA). The two elevated CO_2_ treatment levels were chosen to be close to those predicted for the worst case scenarios for the end the present century and the beginning of the next one [44].

### Carbonate System Determination in the Equilibrated Seawater

The pH measurements were made in a closed 25 ml cell thermostatically controlled at 25°C using a Metrohm 713 pH meter (input resistance >10^13 ^Ohm, 0.1 mV sensitivity and nominal resolution 0.001 pH units) and a glass combined double junction Ag/AgCl electrode (Metrohm model 6.0219.100) calibrated with 8.089 Tris buffer [47] at 25°C; pH values are therefore reported on the total hydrogen ion scale [47]. Temperature and salinity were measured using an Ocean Seven 305 Plus CTD. Total Alkalinity was measured using the method of [48]. The pH, AT and hydrographic data were used to calculate the rest of carbonate system parameters (*p*CO_2_ and DIC) and the saturation stage of Omega Aragonite using CO_2_SYS software [49] set with Mehrbach solubility constants [50] refitted by Dickson & Millero [51].

### Self-righting and Survival in Wild Individuals

To determine whether self-righting ability was associated with predation risk and survival, 21 individuals of small (8.27±0.53 mm) *C. concholepas* were randomly allocated to 1 of 7 independent plastic containers (3 individuals per container). Each container included one individual of *C. concholepas* placed in the normal position and allowed to crawl freely. The other two individuals were exposed in the bottom of the container in an upside-down position; one individual fixed in position with glue on the bottom of the container (unable to self-right) and the other individual not fixed and therefore able to self-right. Each of the 7 containers also contained an individual of the predatory crab *A. hassleri*. The same experiment was then repeated using medium (19.90±1.30 mm) sized individuals of *C. concholepas,* thereby using 21 individuals in each size range. The crabs were left in the containers for 20 min during which time all interactions between the crab and the individuals of *C. concholepas* were recorded. This experiment was conducted on individual locos shortly after they were collected from the field; therefore they were not reared under contrasting levels of *p*CO_2_ and were considered as wild individuals.

### Self-righting in Wild Individuals, in the Absence of a Predatory Cue

Thirty individuals of *C. concholepas* were collected from the field and returned to the laboratory. Four days after collection, self-righting success and self-righting times were evaluated in the absence of any predatory cue. Each individual was placed in a small Plexiglas chamber immersed in a water bath to maintain the temperature ca 12±1°C. Each individual was placed in the chamber for 5 minutes of acclimatization and then the snail was placed upside down in the middle of the chamber. Self-righting time was defined as the time needed by the individual to completely return to its normal upright position. A digital stopwatch was used for measurements, allowing a maximum of 1.5 h to asses self-righting per individual. The total time elapsed from the moment that the individuals were placed upside down to the return to the normal upright position (self-righting time) was measured.

### Growth

To assess the effect of the three different *p*CO_2_ levels on snail size and weight, individuals were randomly selected (n = 10 per treatment) and measured at regular intervals during the acidification phase (0, 11, 45, 52 62, 73 and 83 days of rearing). The size (peristomal length, mm) as a function of *p*CO_2_ treatments were obtained by measurements conducted with a digital calliper. However, shell weight of live individuals (buoyant weight in g^1^) as a function of *p*CO_2_ treatments were measured following the non-destructive technique based on the buoyant weight method [52] with an analytical balance (Adam AFA180 LC).

A parallel exposure was conducted to determine whether any of the 3 seawater treatments used in the acidification experiment was corrosive to the naked shell material. Fifteen empty shells of juvenile individuals (ca. 2.5 cm) were randomly allocated to each of the 3 *p*CO_2_ treatments (5 shells per treatment). The shells were individually placed within 0.5 L plastic containers which were kept semi-immersed in a water bath with running seawater to maintain the temperature ca. 12±1°C. The shells were then exposed to one of the three experimental *p*CO_2_ levels for a total of 30 days. Shell weight as a function of *p*CO_2_ levels was measured at the first day and then again at the end of the 30 day exposure period.

### Self-righting and Predation Risk in pCO_2_ Treated Individuals

**Figure 1:**
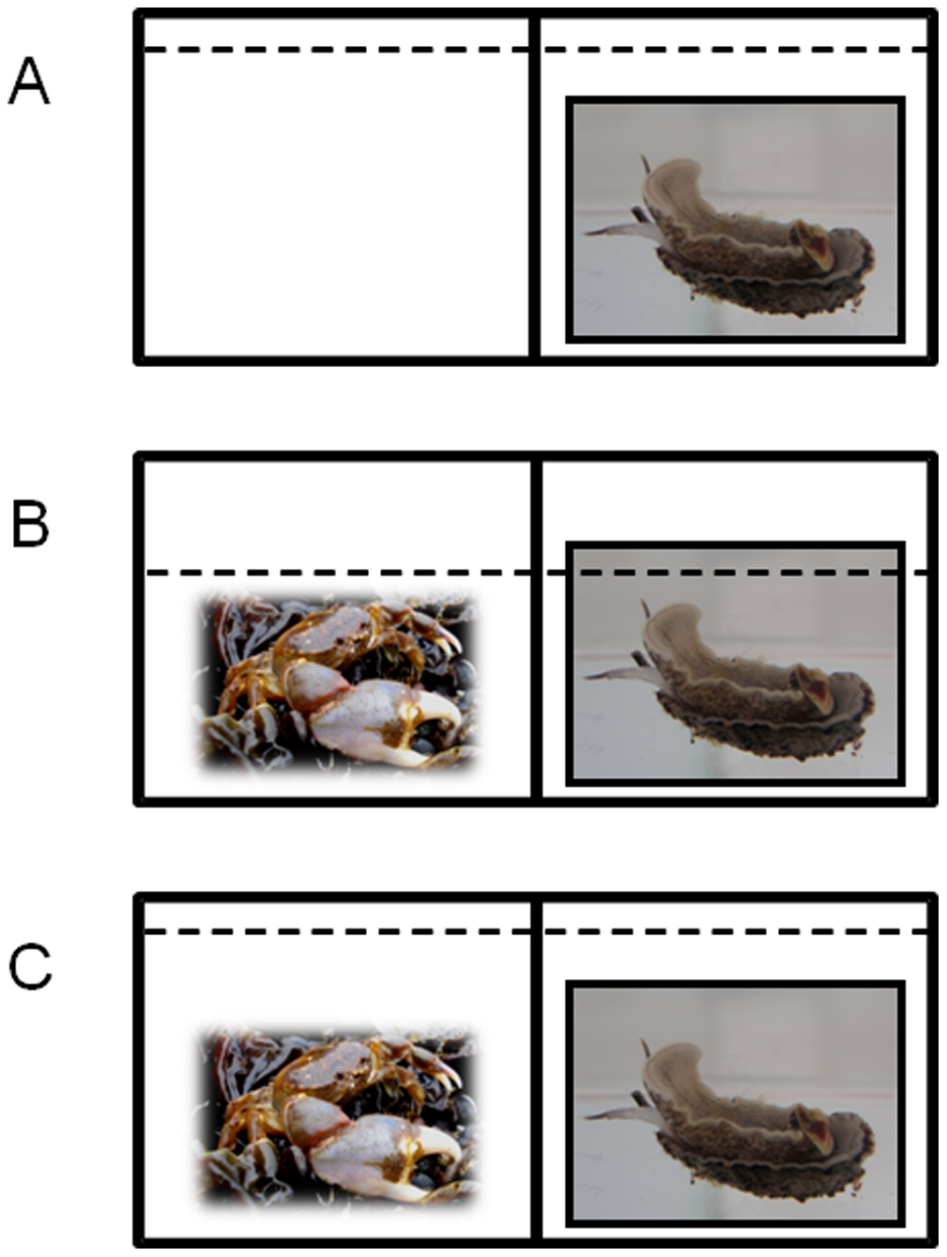
Schematic representation of the three predation risk conditions used to examine the effect of crabs on self-righting in individuals of *Concholepas concholepas*. (A) absence of predatory crabs; (B) presence of predatory crabs and no crab cues and (C) presence of predatory crabs and crab cues. The segmented line represents the level of the seawater contained in the clear Plexiglas allowing or preventing the crab cues from entering the black Plexiglas side of the chamber containing the overturned experimental individuals.

At the end of the acidification phase, both self-righting times and self-righting success were measured in 10 randomly selected individuals from each of the 3 CO_2_ treatments using similar methodologies as those described above for wild individuals. The only difference in the approach used for wild individuals compared to that used for treated individuals was related to the number of individuals available for measurement. For wild individuals it was possible to collect a large number of individuals so that all observations could be made independently on different individuals. However, the acidification phase only contained 10 individuals per treatment so it was therefore necessary to adopt a repeated measures design. Therefore, to assess self-righting times, each individual was sequentially exposed to each of the three predator treatments (described below). Special care was taken to allow the individuals one day of rest between consecutive measurements (i.e., a repeated measures design). If individuals took longer than 1.5 h to turn over, the timing was stopped and they were categorized as either “motionless individuals” or as “with active attempts to self-right” (i.e., extension of the propodium). All measurements were conducted in a clear Plexiglas chamber with two contiguous compartments separated by a plastic mesh. The chamber was immersed in a water bath to maintain the temperature ca 12±1°C, average seawater temperature over the rearing period. Thirty minutes before the measurements were due to be taken the individuals were removed from their exposure containers, placed in similar containers but deprived of food. After this period of acclimatization the individuals were removed sequentially from the containers and placed in the middle of a black Plexiglas chamber located in one of the sides of the clear Plexiglas chamber. In the first treatment the seawater level in the clear Plexiglas chamber was 0.1 cm higher than in the black chamber allowing the free contact between the seawater contained in both chambers (Treatment 1, Fig. 1a). In the second and third experimental treatments an individual of the predatory crab *A. hassleri* was placed in the other compartment. However, as a control for cues associated with crab presence the seawater level in the clear Plexiglas chamber was 0.5 cm lower than in the black chamber preventing the free access of crab visual and chemical cues (Treatment 2, Fig. 1b). In the final treatment the water level was maintained high to allow free contact between the seawater contained in both chambers (Treatment 3, Fig. 1C). To avoid disturbing the experimental individuals during the timing of self-righting, a periscope was used to make the observations. To avoid the confounding effect of using aged seawater or seawater with a different contact time with the predatory crabs, after each trial the chambers were cleaned and filled with fresh seawater allowing 15 min of contact between the crabs and the seawater before the snails were placed in the chamber. Seawater used in the trials was taken from the seawater acidification unit (described previously) so during the righting measurements each individual was held in seawater with the same treatment level of *p*CO_2_ as it had been exposed to during the acidification phase of the study.

### Metabolism

After all self-righting and predation risk observations had been made, all individuals were returned to their exposure containers and the acidification phase continued for a further 17 days. After this additional time each individual had received a total exposure time of 100 days. At this point the individuals were transferred to either a Hamilton® precision syringe (10 ml) used as a respirometry chamber connected to a Hamilton septum sealed with 1 mm silicone dots [53] or, if the individual was larger than 1.0 cm in size, a sealed Plexiglas chamber (30 ml) similar to that of other studies [54], [55] was used. The oxygen consumption of each individual was determined during two consecutive 45 minute closed incubation (data from the first 10 minutes was not used to minimise the impact of potential handling stress). Care was taken to allow the individuals three day of rest between the consecutive incubations and starved them for 24 h before the measurements of oxygen consumption.

Oxygen concentration in the incubation chambers was determined using an optic fibber oxygen-meter (MICROX TX3, PRESENS, GEN), which was located inside the sealed respirometry chambers. All measurements and calibrations were conducted with artificial seawater (salinity: 31 psu, Instant Ocean© sea salt dissolved in distilled water) at the rearing temperature (12±1°C) using an automated water bath (JioTech® Co). The fiber oxygen meter was calibrated before each measurement using a solution of sodium sulphite and aerated seawater respectively. Because stirring affected the snail behaviour it was not included in our methodologies to keep the seawater in the chamber well mixed. Background respiration was determined from control experiments in which no individuals were presents in both types of chambers and oxygen consumption rates were adjusted accordingly, though the background rates were negligible and similar in both chambers. Oxygen consumptions (mg) were standardized by unit of time (h), volume (l) and wet weight (g).

### Statistical Analyses

Chi-squared tests were used to compare the effect of *p*CO_2_ on occurrence of self-righting. One-way ANOVA was used to compare size and weight at the beginning of the experiments, and to compare metabolism, weight and size after the acidification period. Repeated measures analyses with nesting in a main factor (*p*CO_2_ treatment) were performed in a General Linear Model (followed by pairwise comparison using Tukey test between main factors) to test for differences in the self righting time of *C. concholepas* incubated at a given level of *p*CO_2_ in seawater (between subjects) and then confronted with increased levels of predation risk (within subjects repeated measurement nested within *p*CO_2_ treatments) using MINITAB v 14 (MINITAB Inc. 2003) [56].

## Results

### Self-righting and Survival in Wild Individual

Regardless of the size-range, the predatory crab *Acanthocyclus hassleri* exclusively attacked and consumed those wild individuals of *C. concholepas* that were prevented from self-righting. No lethal attacks were observed on those individuals with normal orientation. Only one lethal attack was observed on an individual that had been overturned and then allowed to self-right. The chi-squared test verified that the survival of small size (chi-squared test, χ^2^ = 42.00, DF = 2, P<0.05) and medium size snails (chi-squared test, χ^2^ = 40.05, DF = 2, P<0.05) was not independent of the initial position of the individual of *C. concholepas*.

### Self-righting in Wild Individuals

At the start of the acidification phase of the experiment, no significant differences in self-righting times were found between individuals assigned to the different *p*CO_2_ treatment groups (F_2, 27_ = 0.41; P = 0.666). After being turned over, all individuals were able to successfully self-right, taking on average 13.23 min (±0.92 SE).

### Size and Growth

At the beginning of the experiment no significant differences were detected in the peristomal length (F_2, 27_ = 1.96; P = 0.160), wet weight (F_2, 27_ = 1.53; P = 0.236) and buoyant weight (F_2, 27_ = 1.52; P = 0.237) between the snails (Table 2). At the end of the acidification phase (the 83 day exposure period) no significant differences were detected in the peristomal length (F_2, 27_ = 1.02; P = 0.373), wet weight (F_2, 27_ = 1.45; P = 0.253), and buoyant weight (F_2, 27_ = 1.30; P = 0.290) between the snails reared under differing *p*CO_2_ levels (Table 2). At the end of the acidification phase no significant differences were detected in the shell weight of empty shells (F_2, 12_ = 0.04; P = 0.958). Similarly, no significant differences were detected in the weight of the empty shells of *C. concholepas* (F_2, 12_ = 0.04; P = 0.962) at the end of the acidification phase of 30 days.

**Table 2:** Body size and weight (average ±1SE) of individuals and empty shells of *C. concholepas* reared under different *p*CO_2_ treatments.

Experimental treatments (average levels of *p*CO_2_ µatm), live individuals	*N*	Initial size, mm	Final size, mm
388	10	16.99 (1.87)a	21.59 (0.62)^a^
716	10	18.55 (1.53)a	22.62 (0.57)^a^
1036	10	17.66 (1.89)a	23.03 (0.94)^a^
	***N***	**Initial wet weight, g**	**Final wet weight, g**
388	10	1.0288 (0.3283)^a^	1.6538 (0.0945)^a^
716	10	1.2766 (0.3309)^a^	1.9070 (0.1049)^a^
1036	10	1.1178 (0.3044)^a^	1.8836 (0.1434)^a^
	***N***	**Initial buoyant weight, g**	**Final buoyant weight, g**
388	10	0.3285 (0.1160)^a^	0.5460 (0.0312)^a^
716	10	0.4075 (0.0938)^a^	0.6275 (0.0327)^a^
1036	10	0.3515 (0.1014)^a^	0.6071 (0.0459)^a^
**Experimental treatments (average levels of ** ***p*** **CO_2_ µatm), naked shells**	***N***	**Initial dry weight, g**	**Final dry weight, g**
388	5	1.2991 (0.1639)^a^	1.2898 (0.1630)^a^
716	5	1.2667 (0.1121)^a^	1.2553 (0.1106)^a^
1036	5	1.2504 (0.0915)^a^	1.2391 (0.0907)^a^

Live individuals were exposed for 83 days and fed *ad libitum* with mussels. Differences among levels of *p*CO_2_ treatments were not significant for any measurement.

The individuals were obtained in the field and then exposed for 83 (live individuals) and 30 (empty shells) days under the experimental levels of *p*CO_2_. Initial and final sizes, wet, dry and buoyant weight and the corresponding growth, deposition and dissolution rates were compared by one-way ANOVAs and similar superscripts indicate lack of significant differences between treatments (P>0.05). See text for details.

### Self-righting and Predation Risk in *p*CO_2_ Treated Individuals

**Figure 2:**
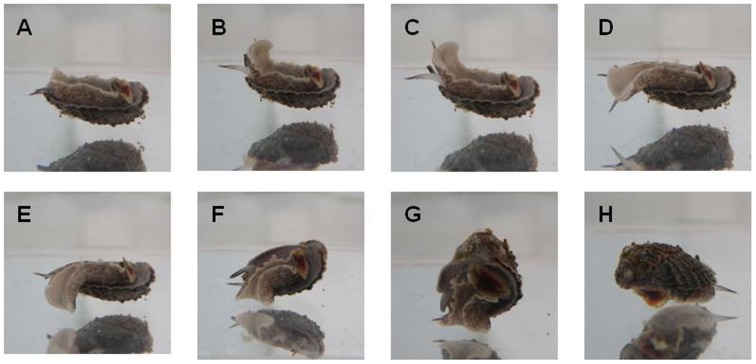
Photographic sequence (A–H) illustrating self-righting behavior in the gastropod *Concholepas concholepas*.

**Figure 3:**
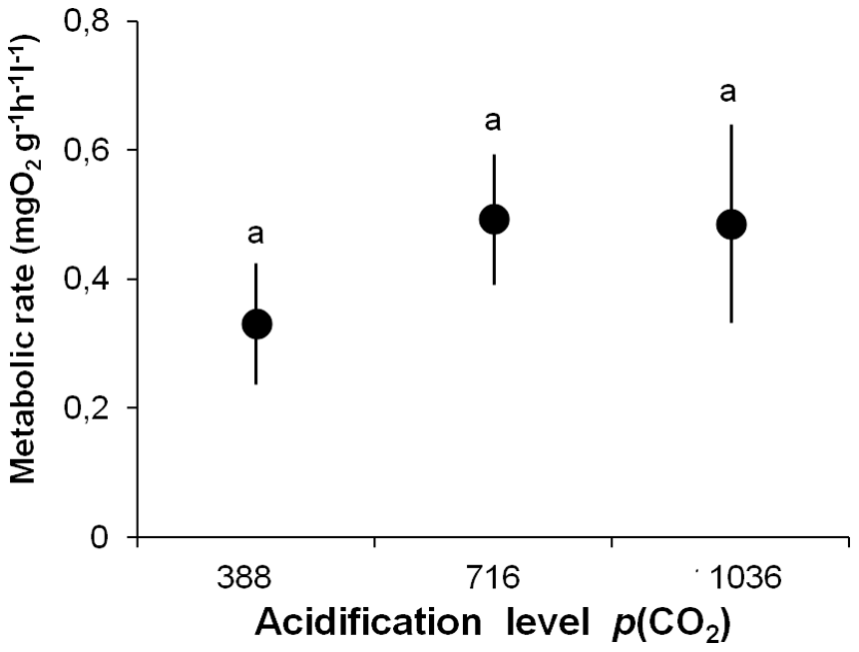
Effect of different *p*CO_2_ (µatm) levels and predation risk on self-righting of *Concholepas concholepas*. Mean (± SE) self-righting time evaluated with no predator (open bars) and with predator cues (filled bars). Where crabs were present there were two contrasting conditions of predatory risk; direct contact between the seawater containing the crabs and the snail individual was either prevented (predator control, gray bars) or allowed (black bars) by modifying the height of the seawater in the Plexiglas chamber. The individuals (n = 10) were reared in the three contrasting pCO_2_ levels and then assigned sequentially to each one of the experimental condition with and without predation risk. Self-righting values that do not differ at 0.05 level in Tukey tests, following a repeated measurement ANOVA, are indicated by a line above the bars.

Self-righting in *C. concholepas*, as in other gastropods [57], involved the extension of the propodium repeatedly until a solid substrate was encountered (Fig. 2). In the absence of a predation risk and regardless of the experimental treatment, it took between 0.5 to 52.2 min for an experimental individual to return to its original position (Fig. 3). Self-righting was faster in individuals reared under increased *p*CO_2_ levels (716 and 1036 µatm) than under normal conditions (388 µatm). On average, self-righting time was three times faster in individuals reared under increased *p*CO_2_ levels than under normal seawater conditions. In the presence of predation risk, self-righting times were shorter than in their absence (Fig. 3), and an entirely motionless quiescent period was never recorded (Table 3). Time taken by *C. concholepas* to reach the upright position decreased with increment of *p*CO_2_ levels (individuals nested within *p*CO_2_ treatments, F_26, 52_ = 11.21, P<0.001), and the differences between pCO_2_ treatments were marginally significant (factor *p*CO_2_ level, between subjects test: F_2, 26_ = 3.12; P = 0.061). However, under different scenarios of predation risk, *C. concholepas* exhibited significantly decreasing self-righting times; the fastest responses were observed in the treatments that included predator cues and slowest in those lacking predator cues (Factor predation risk, within subject test: F_2, 52_ = 17.73; P<0.0001). Differences in self-righting times under predation risk showed the same trend as was seen for increased *p*CO_2_ levels (interaction predation risk x *p*CO_2_ levels, within-subject test: F_4, 52_ = 2.00; P = 0.108). In general terms, when the snails were exposed to predators the self-righting process was always significantly faster with increased *p*CO_2_ levels (388>716 = 1036 µatm CO_2_, Tukey post hoc test; P<0.001 and P = 0.909 for respective pairwise comparisons; however, this result must be interpreted with caution because individual snails are nested within *p*CO_2_ and represent a random factor; Fig. 3). Occurrence of self-righting success showed a significant dependence on *p*CO_2_ treatment exposures (χ^2^ = 10.378; DF = 4; P = 0.035). Maximal self-righting success was only recorded at 1036 µatm (100%, Table 3). However, slightly lower percentages of self-righting were recorded at 388 and 7036 µatm (87.5 and 90.0% respectively; Table 3).

**Table 3:** Concholepas concholepas.

	Experimental treatments (levels of *p*CO_2_ µatm)								
	388			716			1036		
Predatory risk	RS	MP	AA	RS	MP	AA	RS	MP	AA
No risk (no crabs)	87.5	7.5	5	90	0	10	100	0	0
Risk (crab and no water cues)	95.5	0	4.5	90	0	10	100	0	0
Risk (crab and water cues)	100	0	0	90	0	10	100	0	0

Occurrence of individuals (%) displaying self-righting success (RS), motionless posture (MP) or active attempts (AA) under in different conditions of predatory risk and after being reared for 83 days under differing levels of *p*CO_2_.

The same 10 individuals were used in each of the three predator risk trials in four consecutive trials. Motionless individuals were considered those individuals that remain upside down without any evident movement of their propodium during the 1.5 h of observation. Active individuals were considered those displaying active movement of the propodium during a similar period.

### Metabolism

**Figure 4:**
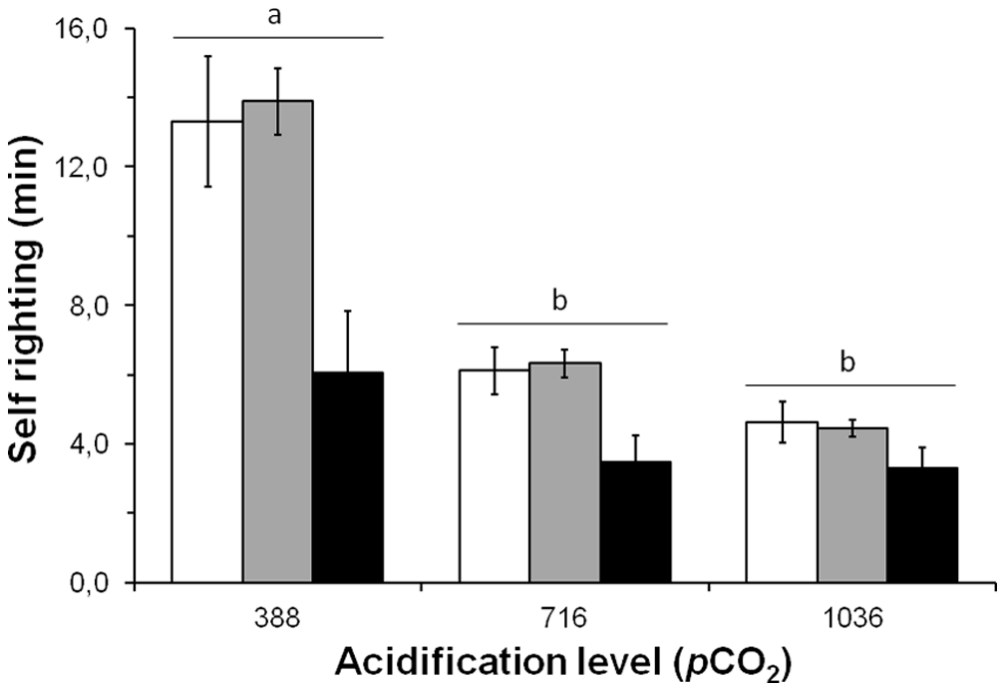
Effect of different *p*CO_2_ (µatm) levels on metabolism of *Concholepas concholepas*. Mean (± SE) metabolic rate after 100 days of exposure to differing levels of *p*CO_2_. The individuals (n = 10) were reared in the three contrasting *p*CO_2_ levels and then assigned sequentially to each one of the experimental condition with and without predation risk. Self-righting values that do not differ at 0.05 level in Tukey tests, following a General Lineal Model, are indicated by a line above the bars. See details in the result sections.

Within each *p*CO_2_ levels, no significant differences between the two consecutive oxygen consumption rates measured in each individual of *C. concholepas* were detected (388 µatm: paired t-test_ = _1.31; DF = 9; P = 0.221; 716 µatm: t-test_ = _0.94; DF = 9; P = 0.374; 1036 µatm t-test = −1.1232; DF = 9; P = 0.249). On average, mean values for oxygen consumption rates were higher in individuals of *C. concholepas* reared at 716 and 1036 µatm CO_2_ compared to those reared at 388 (Fig. 4). However, these differences in metabolism were not significant (F_2, 29_ = 0.62; P = 0.542).

## Discussion

Differences in self-righting times measured in small individuals of *Concholepas concholepas* (ca. 1.5 cm) were detected after being reared for 83 days under differing levels of *p*CO_2_. Faster self-righting was recorded with increased levels of *p*CO_2_ (716 and 1036 µatm). Moreover, this behavioral response to increased CO_2_ levels in seawater interacted with the perception of predation risk in the early ontogeny of *C. concholepas*. Self-righting was only intensified as predation risk increased. In nature, self-righting failure or slow self-righting exposes *C. concholepas* to increased risk of predation after spontaneous dislodgement by wave-swept waters characteristic of rocky intertidal habitats. In the absence of a predator and at increased levels of *p*CO_2_ (i.e. 716 and 1036 µatm CO_2_) this study recorded significantly shorter self-righting times than under currently normal seawater conditions (388 µatm). This study also indicated that small *C. concholepas* that were prevented from self-righting after being turn over and then exposed to the predatory crab *A. hassleri* are lethally attacked. Shorter self-righting times were recorded when *C. concholepas* were in direct contact with cues associated with the same predatory crab. This indicated that self-righting is also modified by perceived risk of predation. Since individuals in their normal position are less susceptible to being attacked, this study highlights the potentially adaptive role played by short self-righting times, as *C. concholepas* improve their chances of survival in the presence of the predatory crab. Ongoing research using y-maze experiments (Manríquez pers. obs) has shown that, *C. concholepas* reared in increased levels of *p*CO_2_ (1036 µatm) from competent larvae to small post-metamorphic sizes (ca. 1.5 cm) failed to detect the position of the predatory crab *Acanthocyclus hassleri*. Therefore, the faster self-righting recorded in the present study and in the presence of the same predatory crab suggests that OA can exert an influence upon two key behavioral traits; the capacity to perceive cues associated with predators and to implement defensive behavior.

In the current study no differences were detected in growth rate measured in terms of size, wet weight, and buoyant weight between treatments at the end of the acidification period, which suggests no net effects of OA on shell calcification in the early ontogeny of *C. concholepas*. Therefore, differences in *C. concholepas* self-righting cannot be attributed to differences in snail size. The absence of effects suggests that this species may be capable of a compensatory mechanism of bio-mineralization in the face of high CO_2_ ocean conditions to avoid net shell dissolution. This may be a possible consequence of the favorable conditions of food availability (*ad libitum*) present during the entire experimental period. To date it is unknown whether shell deposition is affected by pH when feeding is not limited [52]. The use of starved snails to prevent the potential confounding effect of variable feeding rates on growth has been proposed in the literature [4]. Under the experimental conditions of 388 and 716 µatm of CO_2_ the seawater saturation state with respect to aragonite and calcite (see Table 1) was greater than 1 and consequently shell dissolution would not be expected. Our findings suggest that *C. concholepas* have the ability to maintain calcification even at *p*CO_2_ levels of 1036 µatm_,_ when the seawater was saturated for calcite but not for aragonite, and therefore corrosive for the experimental individuals in this treatment. The reduced or absence of negative effects of low saturation states on calcification is in agreement with similar results reported in the literature for other invertebrates [58]–[60]. This suggests that the effects of ocean acidification on net calcification are more complex than expected. Ramajo et al. [61] reported that the external layer and growth edge of the shells of *C. concholepas* juveniles are mainly composed of calcite. Given that calcite saturation states in each of the three *p*CO_2_ treatments used in the current study were greater than 1, shell dissolution would not have been expected. This was indeed that case as shown by an absence of significant differences in shell deposition rate in live individuals or shell dissolution rate of empty shells of juvenile individuals similar to those used in the present study (Table 1). However, a low carbonate saturation state may also affect other developmental rates [62], which were not investigated here. To fully understand the consequences of OA in the early ontogeny of *C. concholepas,* future experimental design should include controlled levels of starvation. Molluscs cover their shells with an organic periostracum, which in small individuals of *C. concholepas* remain in the shell surface even after death. It has been suggested that the periostracum or external organic layers might slow shell dissolution and allow organism to calcify in sweater with reduced pH [58], [60], [63]. This can in part explain the absence of differences found in terms of shell dissolution between live snails and empty shells of *C. concholepas*.

In marine invertebrates, exogenous stressors can have negative consequences on behavior [64]. This impact of stress may be a consequence of a lower ability to conduct physiological functions, and therefore a decreased ability to conduct behavioral functions such as self-righting. In response to stress, animals typically increase their metabolic rate and energy intake [65]. Metabolic rates measured in the present study were in line with average values reported for individuals of *C. concholepas* of similar sizes and in similar seawater temperatures [66]. Moreover, not significant differences were detected between consecutive measurements conducted in a same individual. This suggests that our methodology to measure oxygen consumption was adequate and highlight the time-consistency or repeatability of this trait. The current study found higher, albeit statistically non-significant, metabolic rates at 716 and 1036 µatm *p*CO_2_. Therefore, fast responses to stress obtained at reduced pH during the rearing of *C. concholepas* might represent more optimal pH values for their normal metabolic functions than present pH values. Under the same stressful scenarios (i.e., 716 and 1036 µatm *p*CO_2_) our study found faster self-righting times, which were further reduced by the presence of cues associated with predation risk (i.e. crab effluents). The short self-righting times recorded at those elevated levels of *p*CO_2_ are not consistent with the assumption that stressful conditions may reduce the ability to self-right. During the study *C. concholepas* were fed *ad libitum*, therefore it is possible that all energetic requirements were fully covered. This is supported by an absence of negative effects on growth, survival, and net calcification in CO_2_ exposed individuals. Therefore, we suggest that, during their early ontogeny, exposure to elevated *p*CO_2_ may actually increase the likelihood of *C. concholepas* surviving after being overturned by an exogenous cause. Since gravitational discernment and the adaptive value of the response was not affected, faster self-righting in our study can be attributed to alterations caused by the rearing conditions at different levels of *p*CO_2_. Fast self-righting under increased levels of *p*CO_2_ may reflect an increased anti-predator vigilance stage triggered by stressful conditions associated with OA. Increased avoidance behavior has been also reported in the intertidal gastropod *Littorina littorea* exposed to predatory crabs after rearing under high levels of *p*CO_2_ [67]. This agrees well with the current study and suggests that the consequences of OA may have effects not just at the individual level, but also potentially affect the interaction between predators and their prey. In agreement with studies conducted on other marine calcifying organisms [40], the current study suggests that OA might have no effects on shell growth during the early ontogeny of *C. concholepas*. However, the results presented here also suggest a positive effect on self-righting time during the early ontogeny of the same species. The potential advantage in having fast self-righting after overturning is that it reduces both the duration of the vulnerability to predators and faster contact with the substrate, reducing the chances of being dislodged by waves. Therefore, it is possible that faster self-righting could be a positive consequence of OA in the early ontogeny of *C. concholepas* and other marine invertebrate species in which the turnover response is a common behavioral trait evolved in wave-exposed intertidal zones to avoid predation or wave removal. The Red Queen hypothesis [68] predicts that in predator-prey systems natural selection will favor the co-evolution of predator phenotypes or strategies (i.e. faster attacks) to assure their coexistence with a prey with an improved avoidance behavior (i.e. faster self-righting). Therefore, it is expected that in this eco-evolutionary interaction high prey fitness will induce an increased selection pressure on the predator population that will subsequently evolve. To address this, futures studies are needed that simultaneously expose both prey and their predators to the same changes in their physic-chemical environment.
