# Correction: Ocean Acidification Disrupts Prey Responses to Predator Cues but Not Net Prey Shell Growth in *Concholepas concholepas* (loco)

**DOI:** 10.1371/annotation/f3961ccc-a7e2-442e-9227-ed0bb687450b

**Published:** 2013-07-08

**Authors:** Patricio H. Manríquez, María Elisa Jara, María Loreto Mardones, Jorge M. Navarro, Rodrigo Torres, Marcos A. Lardies, Cristian A. Vargas, Cristian Duarte, Stephen Widdicombe, Joseph Salisbury, Nelson A. Lagos

The images for Figure 3 and Figure 4 are switched.

In Table 1, the header for the last column should be "**1036**" instead of "**136**".

In the References, the citation for Reference 61 is incorrect. The correct citation can be found below:

Ramajo L, Baltanás A, Torres R, Manríquez PH, Lagos NA (2013) Geographic variation in shell morphology of juvenile snails (*Concholepas concholepas*) across the physical-chemical gradient of the Chilean coast. J Mar Biol Assoc UK (in press). 

